# Evaluation of the Association Metformin: *Plantago ovata* Husk in Diabetic Rabbits

**DOI:** 10.1155/2015/167526

**Published:** 2015-10-20

**Authors:** Raquel Díez-Láiz, Juan J. García-Vieitez, M. José Diez-Liébana, Matilde Sierra-Vega, Ana M. Sahagún-Prieto, Ángela P. Calle-Pardo, Nélida Fernández-Martínez

**Affiliations:** Pharmacology, Institute of Biomedicine (IBIOMED), University of León, 24071 León, Spain

## Abstract

In this experimental study we have investigated whether the inclusion of the dietary fiber *Plantago ovata* husk could be recommended as coadjuvant in treatments with oral hypoglycemic drugs. We evaluated the use of *Plantago ovata* husk-metformin association in diabetic rabbits by determining its effects on glucose and insulin concentrations. Six groups of 6 rabbits were used. Groups 1 to 3 were fed with standard chow and groups 4 to 6 with chow supplemented with *Plantago ovata* husk (3.5 mg/kg/day). Two groups (numbers 1 and 4) were used as controls (receiving standard or supplemented chow), two groups (numbers 2 and 5) received metformin orally, and the other two (numbers 3 and 6) were treated orally with metformin and psyllium. Plasma glucose concentrations were lower in groups fed with fiber-supplemented chow whereas insulin levels showed important interindividual variations. Glucose pharmacokinetics parameters showed significant differences in *C*_max_ and *t*_max_ in relation to fiber intake. Insulin pharmacokinetics parameters after treatment with oral metformin showed an important increase in *C*_max_, AUC, and *t*_max_ in animals fed with fiber. We conclude that *Plantago ovata* husk intake can contribute to the oral antihyperglycemic treatment of type 2 diabetes.

## 1. Introduction

The International Diabetes Federation estimated in 2008 that 246 million adults worldwide had diabetes mellitus and the prevalence was expected to reach 380 million by 2025 [1]. This increase in diabetes mellitus results from a rise in new patients of type 2 diabetes, which is a consequence of obesity, an ageing population, lack of exercise, and increased migration of susceptible patients [2].

Although the treatment of diabetes in the 21st century has been dominated by interest in the newer agents (DPP-4 inhibitors, thiazolidinediones), there is still a major role for well-established drugs particularly the biguanide metformin and sulphonylureas [2].

Metformin (dimethylbiguanide) was introduced for the treatment of type 2 diabetes in the 1950s. Nowadays, metformin is included in the list of World Health Organization model list of essential medicines [3]. The antihyperglycaemic properties of metformin are explained by several insulin-dependent and -independent effects that collectively counter insulin resistance and improve glucose homeostasis [4–7]. Also, metformin has actions on body weight [8], blood lipid levels [9], blood pressure [10], thrombosis tendency [11], oxidative stress magnitude [12], inflammation, arterial structure, and vasoprotection [13–15].

*Plantago ovata* husk or ispaghula husk (the husk of the seeds of* Plantago ovata*) is a dietary fiber that induces a mixture of neutral and acid polysaccharides with a rest of galacturonic acid. The polysaccharides are built up of the monomers D-xylose and L-arabinose and ispaghula husk contains 67% pentosans.

This fiber is used under medical prescription but can also be taken by patients on their own initiative in slimming regimens or to regulate intestinal transit being widely employed as a laxative. We cannot forget that in elderly patients constipation is very frequent and type 2 diabetes is more common in this population group.

On the other hand, viscous forms of dietary fiber, like* Plantago ovata* husk, have been shown to improve blood glucose control by trapping ingested carbohydrates inside the viscous gel formed after digestion [16–19]. Due to this fact, it could be recommended as coadjuvant in treatments with oral hypoglycemic drugs [18, 19].

Therefore, the use of metformin and* Plantago ovata *husk at the same time could be beneficial in type 2 diabetic patients.

The aim of this study was to evaluate the use of* Plantago ovata* husk-metformin association in diabetic rabbits. The effect of this association on glucose and insulin levels in diabetic rabbits was determined.

## 2. Material and Methods

All procedures were performed in accordance with the Spanish regulations for the handlings and use of laboratory animals (RD 53/2013). Minimum number of animals and duration of observation required to obtain consistent data were employed.

### 2.1. Animals and Experimental Procedures

To carry out the study, thirty-six healthy New Zealand white rabbits with a body weight range of 2.65 and 3.24 kg were used. The environmental conditions were constant humidity (55 ± 10%), temperature (19 ± 2°C), and 12 h light-12 h dark cycle. The animals were housed in individual metal cages, which allowed the isolation of faeces in a lower container to avoid coprophagia. Rabbits were maintained under these conditions at least 1 week before the assay, with free access to water and chow.

The animals were randomized into six groups of 6 rabbits each. All the animals of the first, second, and third group received standard chow and the rabbits of the fourth, fifth, and sixth group received standard chow supplemented with fiber,* Plantago ovata* husk. This fiber was added to the chow to provide a daily dose of 3.5 mg/kg.

Rabbits were fed with the corresponding chow for two weeks and on day 14, diabetes mellitus was induced by using alloxan. Alloxan (80 mg/kg) dissolved in 10 mL NaCl solution was injected intravenously to the overnight fasted rabbits through their marginal ear vein. Immediately after alloxan, 2 mL of 5% dextrose was intravenously injected and this administration was repeated at 20 minutes and 4, 6, and 8 hours. Three weeks later, on day 35, all the animals of the groups 2 and 5 received 30 mg/kg of metformin by the oral route by gastric intubation, and the rabbits of groups 3 and 6 were treated with* Plantago ovata* husk (300 mg/kg) immediately before metformin oral administration (80 mg/kg). The fiber was administered dispersed in water by gastric intubation. A total of 50 mL water was used for fiber administration and cannula cleaning. The rabbits of the groups 1 and 4 were considered control group of standard and supplemented chow.

After the administration of the corresponding treatment an oral glucose load (3 g) was given to the rabbits and also to the control groups.

The administration of* Plantago ovata* husk included in the chow (groups 4 to 6) was carried out to evaluate the effect of this dietary fiber after its ingestion during a long period of time. Groups 3 and 6 (oral metformin and fiber) served to evaluate the effects of a usual single dose of both compounds (metformin and* Plantago ovata* husk) administered simultaneously. This association was administered to rabbits fed with standard or supplemented chow to evaluate the effects of feeding at the same time.

Blood samples (1 mL) were obtained from the marginal ear vein, using an intravenous catheter, before (time 0) and 30, 60, 120, and 180 minutes after glucose load was given. Immediately after collection, plasma were separated by centrifugation and stored at −20°C until analyzed.

Glucose and insulin were determined in the blood samples. Glucose determination was carried out using a biochemical autoanalyzer (Cobas Integra 400) and insulin by a radiometric method using a kit (Mercodia Ultrasensitive Insulin ELISA, Biosource Europe, SA). The means, standard deviations, and coefficients of variation were computed from the results. Areas under the curve (AUC) were calculated by the trapezoidal rule from plasma glucose and insulin from time 0 until the time of the last sample determined. *C*_max_ and *t*_max_ were directly read from the curves.

### 2.2. Statistical Analysis

The data obtained in the groups of rabbits that received standard chow or supplemented with* Plantago ovata* husk were calculated for each animal, and the data were presented as arithmetic mean ± standard deviation (mean ± SD). Data were analyzed by the Skewness test (to determine normality) and Levene test (to determine uniformity in variance). Data were analysed by two-way ANOVA with a post hoc Duncan test undertaken. A value of *P* ≤ 0.05 was considered significant. All analyses were performed by using SPSS Statistics 21.0 for Windows.

## 3. Results

### 3.1. Glucose

**Figure 1:**
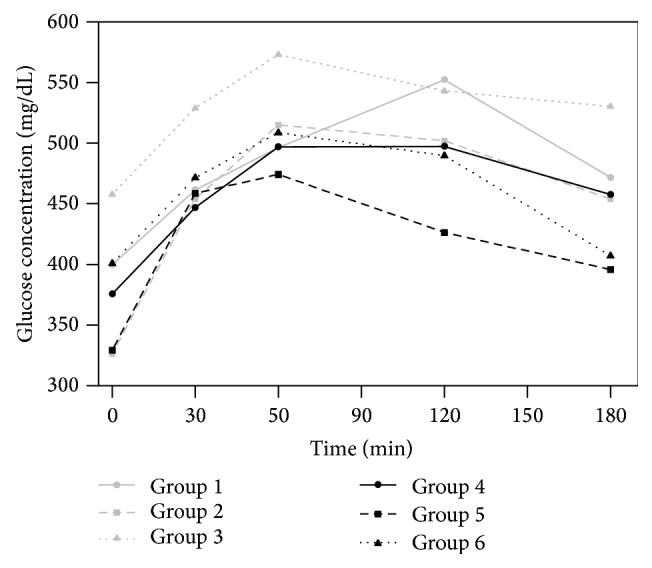
Glucose concentrations after administration of metformin (30 mg/kg) in the 6 groups studied. Group 1: control standard; group 2: oral metformin; group 3: oral metformin with* Plantago ovata* husk; group 4: control supplemented; group 5: oral metformin with supplemented chow; group 6: oral metformin with* Plantago ovata* husk with supplemented chow.

The values of the mean plasma glucose concentrations as a function of time obtained after the oral administration of a 3 g glucose load to rabbits fed with standard chow (groups 1, 2, and 3) or supplemented with* Plantago ovata* husk (groups 4, 5, and 6) are shown in Figure 1.  Table 1 includes the values of *C*_max_, *t*_max_, and AUC determined for glucose in the 6 groups studied.

At time 0, the lower glucose values were found in groups 2 and 5 (oral administration of metformin with standard and supplemented chow): 326.5 and 329.2 mg/dL, respectively. But at the other sampling times, the lower glucose values were determined in group 5 (supplemented chow).

In the control groups, the basal glucose levels were slightly higher (399.9 mg/dL) in the group fed with standard chow (group 1) than in those animals that received chow supplemented (group 4) with* Plantago ovata* husk (375.7 mg/dL). Glucose concentrations were also higher in group 1 (control, standard chow) at all sampling times than in group 4 (control, fiber supplemented chow), being the greatest differences found in the two last sampling times.

Regarding the pharmacokinetics parameters for glucose obtained in these two groups, a slight decrease in *C*_max_ and AUC values (7.9 and 3.8%, resp.) was found.

After metformin oral administration (30 mg/kg), the mean basal glucose levels were very similar, 326.5 and 329.2 mg/dL, respectively, in rabbits fed with standard (group 2) and supplemented chow (group 5). Glucose concentrations were also very similar in the two first sampling times, but, after 60 minutes on, levels were much higher in group fed with standard chow (group 2). The presence of fiber in the chow (group 5) reduced the values of *C*_max_ and AUC (7.6 and 7.2%, resp.).

Finally, after metformin (30 mg/kg) with* Plantago ovata* husk (300 mg/kg) oral administration of the basal glucose levels were higher in group 3 (fed with standard chow), 457.7 mg/dL, than in the group 6 (supplemented chow), 400.9 mg/dL. Glucose concentrations were much lower in rabbits fed with fiber supplemented chow (group 6) at all sampling times. We observed that the inclusion of* Plantago ovata* husk in the chow reduced 11.2% the value of *C*_max_ and 12.2% the value of AUC.

The results indicated that in groups fed with chow supplemented with fiber, the pharmacokinetic parameters showed lower values with significant differences in *C*_max_ and *t*_max_ values but not in AUC (ANOVA, *P* ≤ 0.05).

### 3.2. Insulin

**Figure 2:**
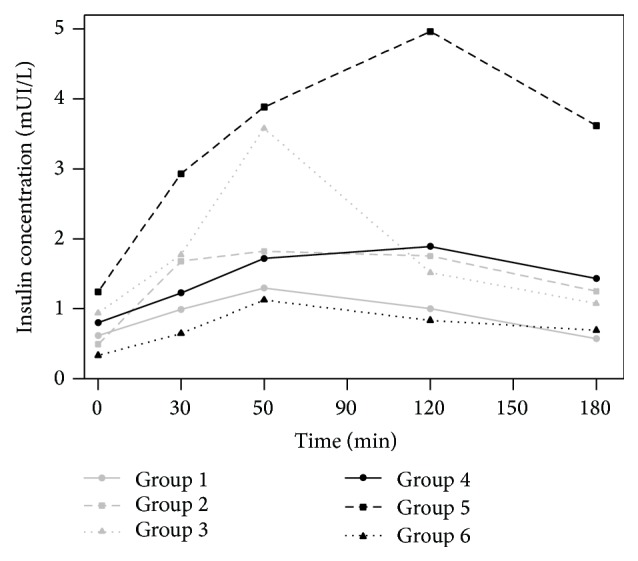
Insulin concentrations after administration of metformin (30 mg/kg) in the 6 groups studied. Group 1: control standard; group 2: oral metformin; group 3: oral metformin with* Plantago ovata* husk; group 4: control supplemented; group 5: oral metformin with supplemented chow; group 6: oral metformin with* Plantago ovata* husk with supplemented chow.

After the oral administration of a 3 g glucose load to rabbits fed with standard chow (groups 1, 2, and 3) or supplemented with* Plantago ovata* husk (groups 4, 5, and 6), the values of the mean plasma insulin concentrations obtained as a function of time are shown in Figure 2.  Table 2 includes the values of the pharmacokinetic parameters *C*_max_, *t*_max_, and AUC determined for insulin in the 6 groups studied.

We observed important interindividual variations in insulin concentrations. Mean baseline values range between 0.492 and 0.936 mUI/L in rabbits fed with standard chow and between 0.333 and 1.242 mUI/L in animal fed with supplemented chow.

The inclusion of* Plantago ovata* husk in the chow increased 28.6% the value of *C*_max_ and AUC was also higher in presence of fiber (38.3%) in the groups of rabbits that were used as control (groups 1 and 3). The values of *t*_max_ decreased from 90 to 80 min in the presence of* Plantago ovata* husk.

The changes caused by the inclusion of fiber in the feeding were very important in those groups (2 and 5), which received oral metformin (30 mg/kg). There was an increase in *C*_max_ (55.2%) and in AUC (58.9%) in animals fed with supplemented fiber chow (group 5). Also, the value of *t*_max_ was modified by the presence of* Plantago ovata* husk, increasing 30 min, from 70 (group 2) to 100 min (group 5).

After oral administration of metformin (30 mg/kg) with* Plantago ovata* husk (300 mg/kg), insulin concentrations were much lower in those animals that also received* Plantago ovata* husk in their feeding (group 6) than in the group fed with standard chow (group 3) at all sampling times. When the chow was supplemented with fiber (group 6), the value of *C*_max_ was a 70.9% lower and AUC 57.5% lower than in rabbits fed with standard chow (group 3). The value of *t*_max_ was very similar in both groups: 60 min for rabbits fed with standard chow (group 3) and 65 min for animals fed with supplemented chow (group 6).

The results showed no significant differences for dietary supplementation with fiber (ANOVA, *P* ≤ 0.05). Regarding the treatment with metformin, there were *C*_max_ between control and oral administration group (Duncan test, *P* ≤ 0.05). Also, we found significant differences in AUC between control and orally metformin administration as well, and between metformin and metformin with* Plantago ovata* husk oral administration (Duncan test, *P* ≤ 0.05).

## 4. Discussion

In this study we evaluated how the inclusion of* Plantago ovata* husk in the chow can modify serum glucose and insulin concentrations in diabetic rabbits in control and metformin group. The effects of the association of a single oral dose of metformin and* Plantago ovata* husk on glucose and insulin levels were also determined.

The results obtained demonstrated that the groups of animals that received chow supplemented with* Plantago ovata* husk showed lower concentrations of glucose than those fed with standard chow. The values of *C*_max_, AUC, and *t*_max_ calculated were also lower in these groups, although only *C*_max_ and *t*_max_ showed significant differences for dietary fiber inclusion.

Several studies have reported that the addition of fiber as a supplement causes an improvement in glycemic control [19–21]. In this way, Díez et al., who evaluated the effects of the inclusion of* Plantago ovata* husk (3.5 mg/kg/day) in diabetic rabbits chow, found significant decreases in glucose concentrations. These authors reported a reduction of 21.7% in *C*_max_ and 26.3% in AUC of glucose concentrations in mild diabetic rabbits and 9.6% in *C*_max_ and 11.2% in AUC in severe diabetic rabbits [20]. In diabetic patients that received psyllium (3.5 g during 6 weeks), Sierra et al. [19] demonstrated that glucose absorption decreased significantly (12.2%) in the presence of fiber.

Our results also indicate that when a dose of* Plantago ovata* husk was administered together with metformin to rabbits fed with chow supplemented with fiber, a reduction in glucose *C*_max_, AUC, and *t*_max_ was observed.

Ziai et al. [21] found similar results in humans. These authors administered 5.1 g of psyllium husk fiber during 8 weeks twice a day half an hour before breakfast and dinner, to patients with type 2 diabetes whose diabetes was controlled with diet plus metformin or glibenclamide. The study showed that after 8 weeks treatment psyllium could reduce fasting plasma glucose and control glucose fluctuations by reducing glycosylated haemoglobin significantly. In addition, in patients treated with metformin, the gastric tolerance of oral antidiabetic was improved.

As we indicated, the largest decreases in glucose concentrations were found in rabbits fed with supplemented chow and that received oral metformin and* Plantago ovata* husk. This result may indicate that* Plantago ovata* husk offers interesting perspectives to be used as adjuvant in patients with type 2 diabetes treated with metformin.

The effects of this dietary fiber on glucose levels have been studied more than the effects on insulin concentrations. There are far fewer studies on the interaction between dietary fiber and insulin levels. In our study, first, we observed a great difference between each rabbit in insulin concentrations. These important interindividual variations were more pronounced in rabbits fed with supplemented chow because baseline values were higher.

The control groups results showed that animals who received chow supplemented with fiber (group 4) exhibited higher concentrations of insulin than rabbits fed with standard chow (group 1). This was confirmed for Díez et al. [20] that reported a significant increase in insulin concentrations for diabetic rabbits fed with* Plantago ovata* husk supplemented chow (3.5 mg/kg/day). This increase was more important in mild diabetic (50.7% in *C*_max_ and 51.7% in AUC) than in severe diabetic rabbits (5.7% in *C*_max_ and 7.4% in AUC). Instead, Sierra et al. [19] observed reductions in insulin levels (5%) in type 2 diabetic patients that received psyllium (3.5 g/4 times a day). Pastors et al. [22] reported a higher reduction; these authors found a significant decrease (12%) in insulin concentrations after the administration of psyllium (3.4 g/twice a day) in type 2 diabetic patients. Also, several authors showed that, in insulin-resistant subjects, dietary fiber may enhance peripheral insulin sensitivity possibly via short chain fatty acids produced by fermentation of fiber in the intestines [23–25]. Fujii et al. [26], found, in 4399 patients with type 2 diabetes, that the insulin secretion index HOMA2%-B was not associated with dietary fiber intake, suggesting that it is unlike insulin secretion induced by increased dietary fiber intake (above 15 g/day for Japanese population) that contributes to improving hyperglycemia.

In our study, the results obtained for insulin were different in the animals that received metformin alone and in those that received metformin and* Plantago ovata* husk at the same time. When metformin administration was in association with* Plantago ovata* husk, insulin concentrations displayed lower concentrations in animals fed with supplemented chow.

So, the pharmacokinetic parameters *C*_max_ and AUC levels decreased in rabbits fed with supplemented chow. These results can be explained due to the fact that fiber can modify intestinal motility and, thus, metformin absorption.* Plantago ovata* husk can also increase the viscosity of gastrointestinal contents and trap the antidiabetic inside the viscous gel formed. Proctor et al. [27] reported that the paracellular transport of metformin was saturable, and perhaps coadministration of metformin and fiber saturated paracellular transport.

This insulin results indicated that the inclusion of* Plantago ovata *husk in the feeding of diabetic rabbits can increase insulin serum concentrations and, therefore, improve type 2 diabetes although no significant differences were found probably due to the great interindividual variations observed.

Our results differ from those of Díaz et al. [28] that evaluated the effect of a dietary fiber from white lupine bran (10 g/day/1 month followed by 20 g/day/1 month) in type 2 diabetic patients treated with oral hypoglycemic drugs. These authors observed no changes in glucose and insulin levels in relation to fiber intake. Flammang et al. [29] studied the effect of a viscous dietary fiber (8.4 g in a single dose/3 days) after an overnight fast in type 2 diabetic adults with oral antihyperglycemic medication. Their results exhibited lower insulin levels unlike those obtained by us, which were increased.

## 5. Conclusions

As tight glycemic control is considered essential in limiting the risk of developing long-term complications of diabetes and taking into account the results obtained in this study* Plantago ovata* husk could have application in improving glucose and insulin control in people with type 2 diabetes.

Although further studies administering metformin with dietary fiber are necessary, we think that* Plantago ovata* husk can contribute to reduce glucose and increase insulin levels in patients with type 2 diabetes in treatment with metformin and, therefore, may be a useful dietary adjunct for the treatment of hyperglycaemia.

## Figures and Tables

**Table 1:** Pharmacokinetic parameters obtained for glucose after the administration of an oral 3 g glucose load in the 6 groups after study. Group 1: control standard; group 2: oral metformin; group 3: oral metformin with *Plantago ovata* husk; group 4: control supplemented; group 5: oral metformin with supplemented chow; group 6: oral metformin with *Plantago ovata* husk with supplemented chow.

	Standard chow			Supplemented chow		
Group	Group 1	Group 2	Group 3	Group 4	Group 5	Group 6
*C*_max⁡_ (mg/dL)	552.5 ± 38.93	533.7 ± 75.11	578.2 ± 25.48	508.9 ± 106.8	493.4 ± 87.92	513.7 ± 72.53
*t*_max⁡_ (min)	130.0 ± 24.49	110.0 ± 45.17	90.0 ± 32.86	80.00 ± 30.98	65.0 ± 58.22	65.0 ± 29.49
AUC (mg·min/dL)	88330.1 ± 5483.5	83147.1 ± 12585.5	96425.1 ± 5524.2	84983.6 ± 21498.8	77194.4 ± 17568.2	84637.1 ± 10774.4

**Table 2:** Pharmacokinetic parameters obtained for insulin after the administration of an oral 3 g glucose load in the 6 groups after study. Group 1: control standard; group 2: oral metformin; group 3: oral metformin with *Plantago ovata* husk; group 4: control supplemented; group 5: oral metformin with supplemented chow; group 6: oral metformin with *Plantago ovata* husk with supplemented chow.

	Standard chow			Supplemented chow		
Group	Group 1	Group 2	Group 3	Group 4	Group 5	Group 6
*C*_max⁡_ (mUI/L)	1.424 ± 0.808	2.279 ± 1.439	3.905 ± 2.139	1.995 ± 1.216	5.082 ± 2.552	1.138 ± 0.643
*t*_max⁡_ (min)	90.0 ± 32.86	70.0 ± 40.99	60.0 ± 32.86	80.0 ± 30.98	100.0 ± 30.98	65.0 ± 29.50
AUC (mUI·min/L)	174.5 ± 60.09	282.6 ± 205.6	342.7 ± 144.2	282.6 ± 178.9	687.6 ± 349.5	145.7 ± 65.20
